# Quantitative Electroencephalography in Guiding Treatment of Major Depression

**DOI:** 10.3389/fpsyt.2018.00779

**Published:** 2019-01-23

**Authors:** Mark J. Schiller

**Affiliations:** ^1^Mind Therapy Clinic, San Francisco, CA, United States; ^2^MYnd Analytics, Inc., Mission Viejo, CA, United States

**Keywords:** electroencephalography, quantitative EEG, biomarkers, depression, machine learning, PEER

## Abstract

This paper reviews significant contributions to the evidence for the use of quantitative electroencephalography features as biomarkers of depression treatment and examines the potential of such technology to guide pharmacotherapy. Frequency band abnormalities such as alpha and theta band abnormalities have shown promise as have combinatorial measures such as cordance (a measure combining alpha and theta power) and the Antidepressant Treatment Response Index in predicting medication treatment response. Nevertheless, studies have been hampered by methodological problems and inconsistencies, and these approaches have ultimately failed to elicit any significant interest in actual clinical practice. More recent machine learning approaches such as the Psychiatric Encephalography Evaluation Registry (PEER) technology and other efforts analyze large datasets to develop variables that may best predict response rather than test a priori hypotheses. PEER is a technology that may go beyond predicting response to a particular antidepressant and help to guide pharmacotherapy.

## Introduction

Psychiatry largely remains unique in the field of medicine in the lack of physiologically based diagnostic tools to diagnose a specific disorder. There are no objective physiology-based tests in psychiatry equivalent to those commonly used in other areas of medicine such as x-ray, ultra sound, or blood tests. There is also no physiological test to guide treatment, comparable, for example, to assaying malignant breast tissue for the presence of estrogen receptors to support treatment with tamoxifen.

This lack of physiological tools to diagnose and guide treatment is of particular concern in the treatment of Major Depressive Disorder (MDD)—a leading cause of disability worldwide. The 2016 National Survey on Drug Use and Health found that 16.2 million adults in the United States had experienced at least one major depressive episode. This number represented 6.7% of all U.S. adults (1). Worldwide the WHO estimated that 4.4% of the population suffered with depression (2). The financial costs of depression are tremendous with the global costs per year of depression and anxiety estimated to be $1.15 trillion (3).

The financial and personal burdens of depression could be reduced by more effective treatment. The Sequenced Treatment Alternatives to Relieve Depression (STAR^*^D) study, a very large, NIMH-funded study of depression treatment algorithms, reported remission rates of 36.8% in Step 1 which dropped to 13% by Step 4 (4). However, a variety of factors, including lack of consideration of dropout rates, may have actually inflated these relatively poor STAR^*^D outcomes (5).

Identifying effective biomarkers to support more effective treatment of depression is extremely important. There has long been interest in the potential for electroencephalography to develop clinically useful biomarkers given the relatively low cost and widespread availability of the technology. Indeed, there have been efforts to identify EEG biomarkers for depression for over four decades.

There are already excellent, comprehensive reviews of this literature (6–8). While some of the reviewed studies examine Event-Related Potentials, the majority of the studies utilized resting state EEGs, evaluating both pre and post-treatment changes and the differences between antidepressant treatment responders and non-responders. These studies identified a number of interesting findings including alpha band changes, theta QEEG cordance, and EEG source localization. More recently, machine learning approaches to identify the most relevant potential biomarkers have shown promise. This paper will first present a few of the most significant studies related to EEG biomarkers in depression and contrast this with a potentially more useful approach utilizing machine learning.

## Alpha Band Activity

Alpha waves have a frequency of ~8–12 Hz, varying slightly based on different definitions. They are generally thought to reflect a relaxed state and are more prominent with closed eyes. A number of studies have generally found elevated alpha power in depressed patients though others found decreased frontal alpha power in comparison to controls and others didn't find any correlations at all (8–10). Some studies found a correlation between alpha excess occipitally and on the right side and antidepressant response (11) though a later study failed to replicate these asymmetry findings (12). This latter study had examined alpha differences between responders and non-responders to treatment with SSRIs and dual-action SSRI/SNRI antidepressants and found that a classifier based upon the median alpha for healthy controls demonstrated good positive predictive value (93.3) and specificity (92.3). However, sensitivity was low (50%) so that half of responders had alpha below the control median and were not expected to be responders.

The studies looking at alpha band activity were hampered by being small, non-randomized treatment of different medications that were not designed to examine medication from non-medication treatment effects (7). However, a more recent study did at least address the small size and non-randomized medication treatment of prior studies in the large, 1,008 subject International Study to Predict Optimized Treatment of Depression (iSPOT-D) in which study subjects were randomized to treatment with either escitalopram, sertraline, or venlafaxine XR. One component of the study was analysis of the predictive effects of alpha band power (13). Neither occipital nor frontal alpha was associated with treatment outcomes at 8 weeks nor did patients and healthy controls differ in occipital or frontal alpha or alpha asymmetry. The study did find a sex difference with relatively greater right frontal alpha in women associated with a good response to the selective serotonin reuptake inhibitors of escitalopram and sertraline, while finding no such effect for the dual action selective serotonin and norepinephrine reuptake inhibitor venlafaxine XR.

## Theta Band Activity

Theta waves have a frequency from 4 to 8 Hz and have also been examined in relation to depression and medication response. Theta activity is related to the activity of the anterior cingulate cortex (ACC). Its affective division in the rostral ACC (rACC) has been found to play roles in assigning emotional valence to internal and external stimuli and emotional expression (14). In his meta-analysis, Pizzagalli found that 19 of 23 studies reviewed suggested that higher pretreatment rACC activity was associated with better treatment response. This link was demonstrated in a variety of different medication and biological treatments suggesting potential in differentiating responders from non-responders in general and not in guiding which particular treatment to use. Pizzagalli and his collaborators examined the role of theta activity in the rACC as part of the Establishing Moderators and Biosignatures of Antidepressant Response for Clinical Care (EMBARC) study in which 248 subjects with usable pretreatment EEG data were randomized to treatment with sertraline or placebo over 8 weeks (15). High rACC activity was found to be a significant predictor of lower Hamilton Rating Scale of Depression (HAM-D) scores at 8 weeks. Baseline rACC activity was responsible for 8.5% of the unique variance in outcome outside of that deriving from clinical and demographic covariates. There was no difference between treatment groups, so that rACC was found to be a general predictor of treatment response rather than a means to guide choice of treatment. This contrasts with the analysis of theta activity from the above-mentioned, large i-SPOTD study which found that both pretreatment high frontal and rACC theta activity were associated with treatment non-response (16). The authors hypothesized that their contrasting results to other studies demonstrating greater response with high rACC theta might be related to different medications studied, differences in degree of treatment resistance, and differences in electrode montages.

The role of theta activity has also been evaluated using cordance, which is a measure reflecting cortical perfusion using a formula that combines absolute and relative power (7). Cordance was investigated in a randomized, double-blinded, placebo controlled study of response to fluoxetine and venlafaxine (17). Decreased frontal theta cordance as measured 1 week after the start of medication treatment correlated with response to these antidepressants at 8 weeks a pattern not seen in placebo responders. Decrease in cordance to predict response and lack of decrease to predict non-response was found to have an accuracy of 72% with sensitivity 69%, specificity 75%, positive predictive value 75%, and negative predictive value 69%.

## Combined QEEG Parameters: ATR

The Antidepressant Treatment Response Index (ATR) is a measure that combines prefrontal theta and alpha power at baseline and Week 1 (7). Specifically it is a non-linear combination of relative alpha and theta power, alpha power in high and low alpha bands, and change in alpha power from baseline to Week 1. ATR is then presented as a probability score from 0 (low probability) to 100 (high probability). In an initial study of ATR, 82 subjects with MDD were treated with either an SSRI or venlafaxine (18). 54.9% of subjects responded based on a ≥50% reduction in HAM-D scores. Retrospective analysis indicated that ATR predicted response with 70% accuracy [82% sensitivity, 54% specificity (*p* = 0.001)].

A second study examined ATR as part of the Biomarkers for Rapid Identification of Treatment Effectiveness in Major Depression (BRITE-MD) study (19). Subjects were treated prospectively with escitalopram 10 mg daily. Response was defined as a ≥50% reduction in HAM-D scores and remission was defined as a HAM-D score of ≤7 after 7 weeks of treatment. The 73 evaluable subjects had an overall response rate of 52.1% and a remission rate of 38.4%. ROC analysis was used to determine a threshold value to maximize classification of responders vs. non-responders. Using this, the ATR predicted response with 74% accuracy, 58% sensitivity, 91% specificity, 88% positive predictive accuracy, and 67% negative predictive accuracy and predicted remission with 74% accuracy, 61% sensitivity, 82% specificity, 68% positive predictive accuracy, and 77% negative predictive accuracy. Of a variety of markers of response investigated, only ATR (*p* = 0.001) and the change in HAM-D at Week 1 (*p* = 0.034) significantly predicted response. The latter measure, it should be noted, is certainly easier to obtain, though at the same time only ATR predicted remission (*p* = 0.002).

A further examination of ATR in a larger subset of the BRITE-MD study was based upon a randomization of subjects at Week 1 to either continued treatment with escitalopram, switch to bupropion XL 300 mg daily, or the combination of escitalopram and bupropion (20). Two hundred and twenty subjects were evaluated at Week 7. Overall the ATR showed 74% accuracy in predicting response and remission (*p* = 0.001 for both). Response rates of those with high ATR values compared to low values were 68 v. 28% (*p* = 0.001). Interestingly, those with low ATR values who were switched to bupropion were 1.9 times more likely to respond than those who remained on escitalopram (53 v. 28%, *p* = 0.034). This is important in demonstrating some potential to differentiate between antidepressant treatments albeit not until 1 week after the initiation of SSRI treatment. The ATR index did not prove useful in predicting response to combination treatment.

## Machine Learning Approaches

The approaches taken above are all based upon a priori hypotheses of specific frequency band variables or combinations thereof. A different approach is to use intensive computational analyses of large datasets to derive predictive biomarkers, a method that has become increasingly common in biomedical research. One pilot study examined responders to SSRIs (noted to generally be sertraline hydrochloride) in 22 subjects with MDD (21). All but one had comorbid diagnoses, and all had failed at least two previous adequate trials of various antidepressants. Pre-treatment EEGs were collected after 10 days of medication withdrawal. Responders were defined as those having ≥30% reduction in HAM-D scores rather than the more common 50% bar. A machine learning prediction method was used to identify candidate features with a Fisher discriminant ratio being used to identify the most relevant features. The most relevant features were primarily found to be a variety of frontotemporal coherence measures in frequencies lying in the low beta frequency band (12–20 Hz). A multi-dimensional model demonstrated an average prediction rate of 87.9% (80.93% specificity and 94.86% sensitivity). There was considerable sex imbalance between responders and non-responders in this study with 6 of 11 females but only 1 of 11 males found to be responders. The model has not yet been tested on a larger dataset and provides no aid in predicting response to non-SSRI antidepressants. The potential for greater applicability would require examining larger datasets and assessing the predictive model for other antidepressant types.

Bailey et al. (22) also used machine learning techniques in attempting to identify responders from non-responders to rTMS treatment. Fifty subjects with treatment resistant MDD and 21 healthy controls had baseline QEEGs. Forty two MDD subjects also had EEG testing at Week 1 and at the end of 5–8 weeks of rTMS treatment. Responders showed lower Montgomery-Asberg Depression Rating Scale (MADRS) scores at Week 1 and endpoint (*p* < 0.01 for both) and lower Beck Depression Inventory (BDI) scores at Week 1 (*p* = 0.03) and endpoint (*p* < 0.01). In an a priori analysis, theta connectivity averaged across baseline and Week 1 was higher in responders than non-responders (*p* = 0.0216). Subsequently, 54 EEG features were chosen to design a predictive model using a linear support vector machine (SVM). The algorithm was evaluated using 5,000 repeats of five-fold cross validation. Using the resulting model, responders could be distinguished from non-responders with mean sensitivity of 0.84 (*p* = 0.001) and mean specificity of 0.89 (*p* = 0.002). However, the relative success of the easily collected MADRS and BDI makes one question the clinical utility of the more difficult to obtain EEG data. In addition, the study was limited by the low response rate of 12 of 50 subjects.

Mumtaz et al. (23) also developed a predictive model of response using a machine learning approach. Thirty four patients diagnosed with MDD were washed out of medications for 2 weeks and then had baseline and weekly EEGs during 6 weeks of treatment with an SSRI. Seventeen subjects were found to be responders based upon a >50% improvement in the BDI. The authors used a wavelet transform analysis to develop an EEG data matrix. This matrix dimensionality was reduced using rank based feature selection. Resulting training and testing led to the development of a logistic regression model which was then validated with 100 iterations of 10-fold cross validation. Frontal and temporal delta and theta frequency variables were found to be the most accurate predictors of response. The model's sensitivity was 95% (±4.3) with a specificity of 80% (±8.8). As in the other machine learning studies the potential for greater applicability would require examining larger datasets and determining utility in predicting response to other classes of antidepressants.

## Meta-analysis of EEG Prediction of Treatment Response

Widge et al. (24) sought to quantify the reliability of QEEG in predicting response to depression treatment in a recent meta-analysis. The authors analyzed articles of interest published between January 2000 and November 2017. Seventy six studies comprising 81 EEG features were identified that merited inclusion for descriptive analysis, while 53 studies comprising 57 EEG features reported sufficient information for inclusion in the meta-analysis. The studies were heterogeneous in degree of treatment resistance, inclusion/exclusion criteria, statistical analysis, EEG methodology, EEG feature studied, and treatment. 57/81 studies looked at medication response while 14/81 investigated rTMS treatment response. Studies tended be small. Quality measures were generally not met with 24 /36 studies testing multiple features failing to make statistical corrections for multiple comparisons and with only 6 of 71 biomarkers reported to have statistical predictive validity subjected to cross-validation. The meta-analysis found an overall sensitivity of 0.72 (95% CI = 0.67–0.76), specificity of 0.68 (95% CI = 0.63–0.73), and log (diagnostic odds ratio) 1.89 (95% CI = 1.56–2.21) indicating that predictive power was greater than chance, though without any difference between biomarkers or treatment type. Funnel analysis suggested a strong publication bias, while further analysis suggested that the predictive power was fueled by small studies with strong positive results. The authors stated that their “results do not imply QEEG findings were not real” but that greater rigor and replication of prior positive studies were necessary for QEEG to be a reliable tool ready for clinical practice. Of note, the meta-analysis did not include results from the EMBARC study, presumably as that study publication fell outside of the stated time parameters, though there was also a mention that insufficient information was provided for inclusion in a meta-analysis.

## Peer

The Psychiatric Encephalography Evaluation Registry (PEER) previously known as referenced-EEG (rEEG) is another machine learning approach which is applied to a large dataset. Currently the PEER database comprises ~11,000 baseline EEGs and 39,000 medication treatment outcome points (25). There are a number of characteristics of PEER that set it apart from other approaches that have been reviewed here. One is that PEER is not a diagnostic tool to predict response to treatment but a tool to guide medication selection. Medication guidance is not restricted strictly to antidepressant treatment; the technology provides indications for a broad range of psychiatric medications. Of note, PEER was specifically not included in the meta-analysis by Widge and his colleagues, as it is not a diagnostic test predicting treatment response but a tool to inform pharmacotherapy (24). PEER has been applied to a range of psychiatric diagnoses in addition to depression. This is based upon a primary assumption that patients with similar EEG biomarkers should respond to the same medications in a consistent manner regardless of diagnosis. This assumption was derived from the observation that psychiatric diagnoses are defined by symptoms rather than objective physiological measures. This contrasts with non-psychiatric medicine, where for example the presence of chest pain and other associated symptoms may point to the presence of a myocardial infarction, but its definition relies on the physiological definition of cardiac cell damage or death, and the diagnosis is demonstrated by physiological tests such as an electrocardiogram or cardiac enzyme assay. Thus, psychiatric patients within a diagnostic category may demonstrate phenomenological similarities but be physiologically heterogeneous and not responsive to the same medications. At the same time patients receiving different diagnoses may have important physiological similarities that would potentially respond to similar medications. A further unusual aspect of PEER is that a patient may simultaneously have different biomarkers that suggest different types of medications resulting in treatment with medication combinations. This seems consistent with general clinical practice in which multiple medications are often prescribed. The use of medication combinations complicates evaluating PEER in comparison to evaluating how well a single electrophysiological measure predicts response to a specific type of medication.

**Figure 1:**
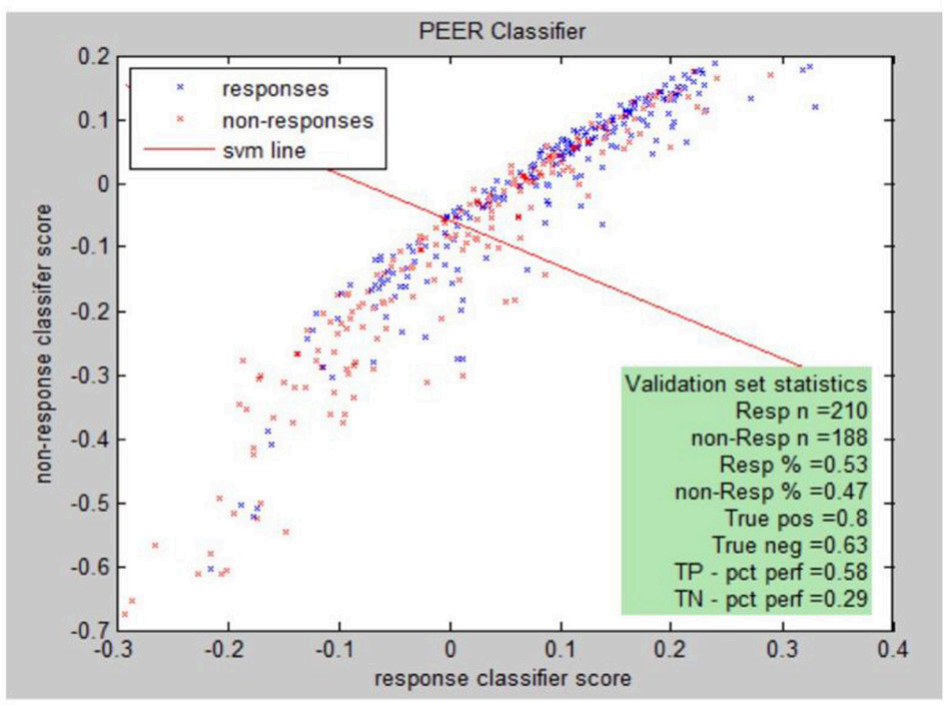
PEER medication response scatter plot.

PEER Interactive, the term describing the report generator, provides a statistical analysis comparing electrophysiological abnormalities (identified by comparison to a normal database of subjects screened for psychiatric and neurological disorders) of a patient to abnormalities found in known responders to a medication in the PEER outcome database (25). PEER Interactive uses two response/non-response classifiers so a scatter plot can be utilized to best represent if a certain subject will be a responder or non-responder to specific medications in the PEER Interactive database (Figure 1). The first classifier (C1) is based upon “net hits” and the second classifier (C2) is based upon a logistical regression.

Responsiveness to classifiers for a specific medication can be presented graphically. The graph illustrates this for classifier I (C1) and classifier II (C2) for the population of either responders or non-responders to the medication. Each point represents: (score on C2, score on C1, response (blue), non-response (red).

Peer interactive uses machine learning techniques to develop combinatorial algorithms with the best predictive power from the full range of available QEEG variables. The individual medication models are tested through multiple cross validations which test the performance of the model against a set of cases not used to develop the model itself. The true positive (complement of type I error) and true negative (complement of type II error) are reported. A validation sample is developed by querying the outcomes database for medication responses not used in constructing the model. The validation sample is further subdivided into a tuning sample and the final validation sample. The tuning sample helps to refine the model by running the scoring model and comparing the score distribution to known responses. The final validation sample is used to further validate the model by running the model without any adjustment of parameters or thresholds. The model is ready for use if the results of the final validation meet the specifications of the previous clinical correlations. The specific machine learning algorithms are proprietary, though drug/class models are reported. In addition, the model is periodically refined as the PEER outcome database is expanded and additional medications are added to this database.

There have been four randomized, controlled trials of PEER technology or its earlier iteration of rEEG. Suffin and colleagues conducted a pilot study on 13 subjects with treatment-resistant major depression who were randomized to a usual care, control group based on the clinical decisions of treating psychiatrists in a naturalistic setting or the experimental EEG-guided medication group (26). Subjects and independent rating physicians were blinded to treatment group. After 6 weeks the decline in HAM-D scale and BDI scores in the experimental group were significantly greater (*p* < 0.009). A larger percentage of subjects in the experimental group had good to excellent outcomes compared to the control group based on Clinical Global Impression-Improvement scores (*p* = 0.02).

Debattista et al. (27) conducted a randomized, blinded, controlled pilot study of treatment-resistant depression in which subjects were treated based upon the Texas Medication Algorithm Project (TMAP) or guided by referenced-EEG. Eighteen subjects completed the pilot study. Outcomes were compared regardless of assigned treatment group based on whether TMAP and rEEG treatments were equivalent after 10 weeks. In comparing the 12 subjects with treatment consistent with the rEEG report vs. the 6 subjects with TMAP guided treatment inconsistent with the rEEG report there was a significantly greater mean change in Quality of Life Enjoyment and Satisfaction Questionnaire (Q-LES-Q) and Quick Inventory of Depressive Symptomatology (QIDS) scores (*p* < 0.0094 and *p* < 0.0066).

This pilot study helped refine the design of a larger, multicenter, randomized, controlled, single-blinded study in relatively treatment-resistant subjects with major depression (28). One hundred and fourteen subjects were randomized to rEEG-guided treatment or to a control group treated based upon a modified STAR^*^D protocol. Eighty two subjects completed the 12 week study. In a per protocol analysis, rEEG-guided subjects had significantly greater mean change improvements in QIDS-SR16 and Q-LES-Q scores (*p* < 0.0002 for both; Table 1). In addition, there was increasing separation on these measures between groups over the course of the study. The rEEG-guided group also showed superiority in 9 of 12 secondary endpoints.

**Table 1:** Least square means.

	**rEEG-guided**	**Control**	***p*-value**	**90% CI**
**PER PROTOCOL**				
QIDS-SR16	−6.8 (SE 0.35)	−4.5 (SE 0.38)	<0.0002	1.52 to 2.99
Q-LES-Q-SF	18.0 (SE 1.06)	8.9 (SE 1.14)	<0.0002	−11.21 to −6.81
**MODIFIED ITT**				
QIDS-SR16	−5.7 (SE 0.30)	−4.2 (SE 0.34)	<0.0002	0.84 to 2.17
Q-LES-Q-SF	14.1 (SE 0.92)	8.0 (SE 1.05)	<0.0002	−8.17 to −4.07

*Adapted from DeBattista et al. (28)*.

*Repeated measures, two tailed, mixed procedure with covariance structure including stratification and study sites in the model*.

Iosifescu et al. (29) reported on the use of PEER Interactive in a study at two military hospitals (Walter Reed National Military Medical Center and Fort Belvoir Community Hospital). This was a prospective, randomized, controlled, single-blinded study of subjects with a primary depressive disorder diagnosis who were not required to be treatment-resistant. One hundred and fifty subjects were enrolled and were randomized to a control group treated per VA/DOD Clinical Guidelines or PEER-guided treatment over 6 months. A *post hoc* analysis analyzed subjects based upon the PEER report being followed (RF) or not being followed (RNF). The percentage reduction in QIDS-SR16 was 144% greater for the RF vs. RNF groups (*p* = 0.029). Reduction in suicidal ideation was 75% greater for the RF vs. the RNF group on the Concise Health Risk Tracking Scale—Self Report (CHRT-7SR) (*p* = 0.0017). There was also a 139% greater improvement in the PTSD Checklist Military/Civilian (PCL/MC) in the RF vs. RNF group (*p* = 0.0348; Table 2).

**Table 2:** *P*-values obtained by ANOVA.

**Endpoint**	**RF %change**	**RNF %change**	**Difference**	***n***	***p*-value**
QIDS-SR16	−30.0	−12	−18%	39	0.029
CHRT	−24	−14	−10.0%	150	0.002
PTSD	−9	−4	−5%	91	0.035
CGS	−23	−13	−10.0%	145	0.017
CGI-physician	−34	−22	−12%	150	0.002
CGI-patient	−40.0	−22	−18%	150	0.0001

*Adapted from Iosifescu et al. (29)*.

*QIDS-SR, Quick Inventory of Depressive Symptomatology Self-Report; CHRT, Concise Health Risk Tracking Scale; PTSD, Post-Traumatic Stress Disorder; CGS, Clinical Goal Setting; CGI, Clinical Global Impressions*.

## Discussion

The studies reviewed here suggest that there are aspects of quantitative EEG that do correlate with response to pharmacologic treatment of depression. While these findings may be of interest in helping us to better investigate the biological underpinnings of depression and provide direction into future avenues of research, ultimately the important question is whether these potential biomarkers have utility in guiding treatment in actual clinical practice. In that the clinical marketplace has primarily indicated that the answer is no. Despite decades of interest in electrophysiological biomarkers, none of the single variable measures reviewed above has generally become accepted on a clinical basis. The combinatorial ATR measure has also been of research interest but has not made its way to general clinical use despite prior significant attempts to commercialize the technology. This lack of success may have resulted from inadequate marketing resources or other purely business factors but may also point to the medical community not finding sufficient value to change clinical practice. Testing to predict whether an antidepressant would ultimately be effective 1 week after already beginning treatment may not be a worthwhile cost without greater predictive accuracy since the important outcome—actual clinical response—will be available after a few more weeks of continued treatment.

Machine learning approaches, however, may prove key in bringing the use of EEG biomarkers from a state of research interest to clinical relevance. PEER technology in particular is being commercialized and is currently used in an expanding number of clinical settings, suggesting clinical value to the effectiveness so far demonstrated in the research projects reviewed. The commercialization model is that of a reference laboratory. Clinicians arrange QEEG testing following standard procedures with any available, local EEG equipment. The resulting data is electronically sent in a secure manner to be analyzed by PEER Interactive. Clinicians are then sent a PEER Report to inform their prescribing decisions in conjunction with clinical factors. In addition to the value of machine learning to derive the most useful predictive markers from large datasets, PEER technology has further advantages in being a pretreatment rather than mid-treatment test and in not being a predictor of response to a single medication or single medication class. Thus, it is a potential tool for broadly guiding effective psychiatric pharmacotherapy of depression. Because of the underlying assumptions, PEER technology is intended to be useful in disorders other than depression, which is the focus of this review. In addition to further research support for the effectiveness of this technology that would benefit from larger studies, further expansion of its current clinical use will be telling.

## Author Contributions

The author confirms being the sole contributor of this work and has approved it for publication.

### Conflict of Interest Statement

The author declares that the research was conducted in the absence of any commercial or financial relationships that could be construed as a potential conflict of interest.
